# Supplementary material for: Clostridioides difficile bile salt hydrolase activity has substrate specificity and affects biofilm formation
Source: NPJ Biofilms Microbiomes. 2022 Nov 30;8:94. doi: 10.1038/s41522-022-00358-0 (PMC9712596; doi:10.1038/s41522-022-00358-0)
Supplement: Supplementary file 1 — Supplementary Material [file 41522_2022_358_MOESM1_ESM.pdf]

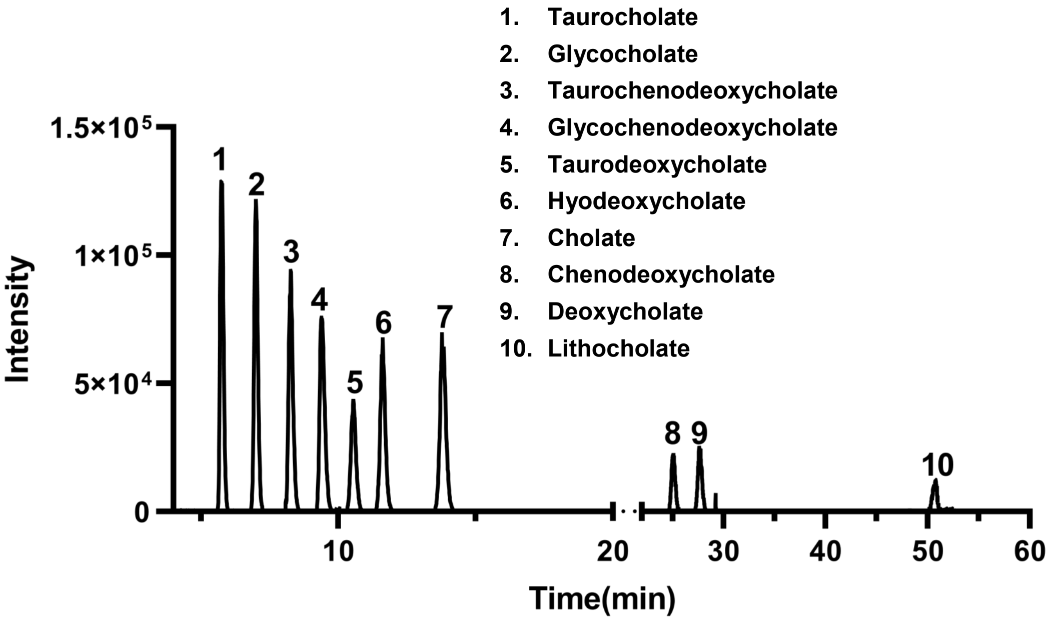

<u>**Supplementary Figure 1. Bile acid standards using HPLC**</u>

Indicated bile acids were separated by High-Performance Liquid Chromatography. Compounds were detected by evaporative light scattering. Standard curves were generated by calculating the area under the corresponding peaks.

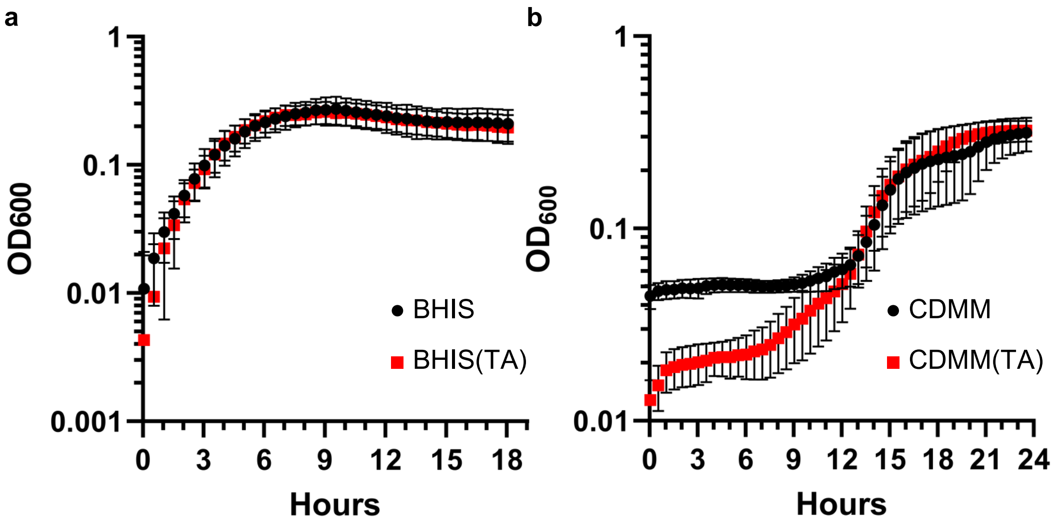

Supplementary Figure 2. *C. difficile* growth is not impacted by taurocholate

A) *C. difficile* UK1 was grown in BHIS medium alone or supplemented with 0.1% taurocholate. B) *C. difficile* UK1 was grown in minimal medium alone or supplemented with 0.1% taurocholate. Values represent the average of three independent experiments and error bars indicate the standard error of the mean. For clarity, every 9th data point is shown. Statistical significance was determined using a 2-way ANOVA with Tukey's test for multiple comparisons (the samples differences are not statistically significant).
